# Composite Face Effect Predicts Configural Encoding in Visual Short-Term Memory

**DOI:** 10.3389/fpsyg.2019.02753

**Published:** 2019-12-11

**Authors:** Lilian Azer, Weiwei Zhang

**Affiliations:** Department of Psychology, University of California, Riverside, Riverside, CA, United States

**Keywords:** visual short-term memory, Gestalt, holistic face processing, receiver operating characteristic, individual difference

## Abstract

In natural vision, visual scenes consist of individual items (e.g., trees) and global properties of items as a whole (e.g., forest). These different levels of representations can all contribute to perception, natural scene understanding, sensory memory, working memory, and long-term memory. Despite these various hierarchical representations across perception and cognition, the nature of the global representations has received considerably less attention in empirical research on working memory than item representations. The present study aimed to understand the perceptual root of the configural information retained in Visual Short-term Memory (VSTM). Specifically, we assessed whether configural VSTM was related to holistic face processing across participants using an individual differences approach. Configural versus item encoding in VSTM was assessed using Xie and Zhang’s (2017) dual-trace Signal Detection Theory model in a change detection task for orientation. Configural face processing was assessed using Le Grand composite face effect (CFE). In addition, overall face recognition was assessed using Glasgow Face Matching Test (GFMT). Across participants, holistic face encoding, but not face recognition accuracy, predicted configural information, but not item information, retained in VSTM. Together, these findings suggest that configural encoding in VSTM may have a perceptual root.

In natural vision, visual scenes often consist of individual items (e.g., trees) and global emergent properties of items as a whole (e.g., forest). These different levels of representations can all contribute to perception (Navon and Norman, 1983; Kimchi, 1992), natural scene understanding (e.g., Greene and Oliva, 2009), sensory memory (Cappiello and Zhang, 2016), visual short-term memory (Brady et al., 2011; Tanaka et al., 2012; Orhan et al., 2013; Nie et al., 2017), and long-term memory (Hunt and Einstein, 1981; Yonelinas, 2002). In addition, there could be significant interactions between these hierarchical representations, for example, enhanced item processing by the global context (Fine and Minnery, 2009; Santangelo and Macaluso, 2013). Despite these various hierarchical representations across perception and cognition, global representations receive considerably less attention in memory research than item representation (e.g., Brady et al., 2011). The present study has thus assessed whether configural information, one kind of global representations, retained in VSTM is related to overall holistic processing in vision.

The representations of global information in VSTM have gained some support in recent years (e.g., Brady and Alvarez, 2015; Nie et al., 2017). For example, experimental manipulation of configural information at retrieval could either impair or facilitate VSTM for item information (Jiang et al., 2000, 2004; Treisman and Zhang, 2006). Specifically, changes in configural context (e.g., by changing features of non-probed items) at test can impair VSTM for spatial locations (Jiang et al., 2004) and non-spatial features (Vidal et al., 2005; Jaswal and Logie, 2011). In addition, manipulation of configural encoding upon formation of VSTM representations can also affect the later access to stored VSTM contents (Delvenne et al., 2002; Xu, 2006; Gao and Bentin, 2011; Gao et al., 2013; Peterson and Berryhill, 2013). For example, surrounding circles on orientation bars can considerably reduce VSTM storage capacity for orientation information (Delvenne et al., 2002; Alvarez and Cavanagh, 2008), presumably because the surrounding circles severely interrupted configural encoding (Xie and Zhang, 2017). It is highly likely that these effects of perceptual organization on VSTM are a natural extension of configural encoding in perceptual processing. Consistent with this hypothesis, Gestalt cues such as connectedness (Woodman et al., 2003; Xu, 2006), similarity (Peterson and Berryhill, 2013), and closure (Gao et al., 2016) could facilitate grouping of individual items during VSTM encoding, leading to increased storage capacity. In addition, the configural superiority effect (CSE) has demonstrated that individuals’ ability to detect a target among distractors is significantly faster in the presence of contextual cues and closure (Nie et al., 2016). In other words, closure of stimuli allows individuals to form Gestalts resulting in rapid detection of the target stimulus and successful inhibition of distractor stimulus. Nonetheless, it is unclear whether holistic encoding, for example configural and holistic encoding as opposed to first-order processing of isolated feature in object and face recognition (Kimchi, 1994; Maurer et al., 2002; Piepers and Robbins, 2012), is related to VSTM for configural information. The present study has thus assessed whether holistic face processing (e.g., Tanaka and Farah, 1993) can predict configural VSTM across participants, using an individual differences approach.

Configural encoding in VSTM was estimated using a change detection task for orientation and a recently developed Receiver Operating Characteristic (ROC) model (Xie and Zhang, 2017). In this task Figure 1A, participants memorized five briefly presented orientation bars in a memory array. Following a 1,000-ms delay interval, participants reported whether an orientation bar in a test array contained a new orientation (“new” response) or the old orientation (“old” response) on a 6-point confidence scale, as compared to the corresponding bar presented at the same location in the memory array. These responses were used to construct ROC curves, the function relating the probability of “old” responses on *old* trials (hit rate) to the probability of “old” responses on *new* trials (false alarm rate) using Signal Detection Theory (Macmillan and Creelman, 2005). The resulting ROCs were then fitted with a Dual-Trace Signal Detection ROC model (DTSD, Xie and Zhang, 2017) to quantitatively assess contributions of item-based encoding (i.e., individual orientation) and configural encoding (i.e., the overall shape of all orientation bars) in VSTM (for details of the model, see Xie and Zhang, 2017).

**Figure 1:**
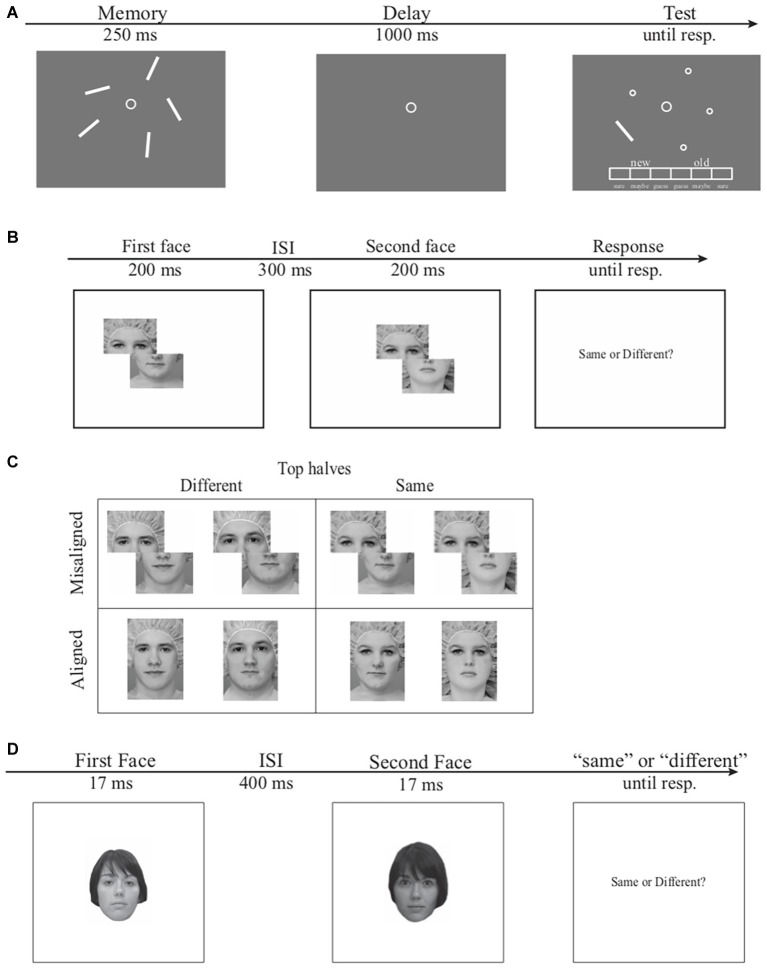
Examples of the stimuli and general procedure. **(A)** The procedure and stimulus for the orientation change detection task. On each trial, a memory array of five orientation bars was presented for 250-ms after a 1,000-ms fixation screen. Following the memory display, a 1,000-ms blank delay interval appeared on screen and a test display was presented until participants reported whether the orientation of the probed bar was the same or different from the orientation of the corresponding memory item on a 6-point confidence scale. **(B)** The Le Grand composite-face task. The first face, which was aligned or misaligned, was presented for 200-ms and followed by a 300-ms interstimulus interval. A second face was presented for 200-ms corresponding to the alignment of the first face. Participants were instructed to respond if the top half of the second face was the same or different as the top half of the first face while ignoring the bottom halves. **(C)** Le Grand composite face stimuli. The top row consists of two face pairs from the misaligned condition and the bottom row consists of two face pairs from the aligned condition. The top halves of the two face pairs are either identical to one another (right panel) or different from one another (left panel). **(D)** The modified GFMT task. The first face was presented for 17-ms and followed by a 400-ms interstimulus interval. A second face was presented for 17-ms and participants were instructed to respond if the second face was the same or different as the first face regardless of difference in visual angel or contrast.

Holistic encoding in face processing was estimated using the composite face effect (CFE) of the Le Grand face task (Le Grand et al., 2001, 2004; Mondloch et al., 2002). Unlike the face inversion task that taps on second-order relational information in face processing, the CFE is a more robust measure of holistic face processing (Maurer et al., 2002). In this task, two brief displays of composite faces were presented sequentially (Figure 1B). Each face consisted of a top half and a bottom half that form a complete face when combined together. Participants reported whether the top halves of the two composite faces were the same (e.g., faces in the right column in Figure 1C) or different (e.g., faces in the left column in Figure 1C) across the two displays while ignoring the two bottom halves which were always the same across the two displays. Although the two bottom halves were completely task irrelevant, they can empirically and phenomenologically interfere with the same/different judgments of the two top halves (e.g., Le Grand et al., 2004), due to holistic face processing that encodes the top and bottom face halves as an integrated face instead of separate face segments. Orthogonal to the manipulation of the same versus different top face halves, the top and bottom face halves were misaligned (e.g., faces in the top row in Figure 1C) on half of the trials and properly aligned (e.g., faces in the bottom row in Figure 1C) on the remaining trials. The interference from the irrelevant bottom face halves tends to be reduced for the misaligned condition, as compared to the aligned condition, because the top and bottom halves from the misaligned condition are less likely to be perceived as an integrated face (Le Grand et al., 2001, 2004). This difference in performance between the aligned and misaligned conditions, the CFE, is thus an operational definition of the interference caused by task-irrelevant bottom face halves.

Overall face discrimination was also assessed, using a two-interval forced choice task (Figure 1D) with face stimuli from the Glasgow Face Matching Test (GFMT, for details see Burton et al., 2010). Participants in this task reported whether the two sequentially presented faces had the same or different identity. This modified GFMT was chosen over other face identification tasks (Bruce et al., 1999) to minimize potential involvement of VSTM (Xie and Zhang, 2017). Specifically, in the Bruce et al. face identification task for example, a target face is matched to one of the 10 simultaneously presented faces. The matching process in this task could involve several eye movements across stimuli, leading to significant involvements of VSTM (e.g., Irwin, 1991).

We hypothesized that holistic encoding in face processing assessed as CFE, but not the overall face matching ability assessed as accuracy in the modified GFMT discrimination task, would predict configural encoding, but not item encoding, in VSTM across participants.

## Methods

### Participants

Forty-six UC Riverside students (31 females) between the ages of 18 and 30 with normal color vision and normal or corrected-to-normal visual acuity participated in this study for course credit. Four additional participants were excluded because they did not complete all three tasks within a 1-h experimental session. The experimental procedure was approved by the Institutional Review Board of University of California, Riverside. All participants were provided written informed consent. *A priori* power analysis (Faul et al., 2009) for *r*-based effect size at a medium level (0.35) suggested that a total sample size of 50 participants would provide 80% statistic power. *Post hoc* power analysis for 46 subjects for a *r*-based effect size of 0.38 yielded 84% statistical power.

### Stimuli and Procedure

All stimuli were presented, using PsychToolbox-3 (Brainard, 1997) for Matlab (The MathWorks, Cambridge, MA), on a LCD monitor with a homogeneous gray background (6.7 cd/m^2^) on a macOS operating system with a refresh rate of 60 Hz at a viewing distance of 57 cm.

The stimuli and procedure were the same as the uncircled bar condition in Experiment 1 of Xie and Zhang (2017). In this VSTM change detection task (Figure 1A), the memory array consisted of five white orientation bars (3° in length and 0.15° in width) in different orientations quasi-randomly selected from 180°circular space. The angular differences between any two orientations were more than 12°. The orientation bars were presented at five locations randomly chosen from eight equally spaced locations on an imaginary circle 4.5° in radius. Each trial began with a 1,000-ms fixation at the center of the screen, followed by a 250-ms memory array of five orientation bars. Participants were required to memorize and retain as many orientation bars as possible over a 1,000-ms blank delay interval. At test, one bar randomly selected from the memory set reappeared at its original location, whereas other memory items were replaced with circles (0.3°) as placeholders. Participants reported whether this bar had the “old” or a “new” orientation as compared to the corresponding item at the same location of memory array. The “old”/“new” decision and the confidence for this decision (e.g., sure new, maybe new, or guess new, sure old, maybe old, or guess old) were reported on a 6-point confidence scale (16.2 by 0.8° in visual angle) presented at the bottom of the screen using a computer mouse by the participants. The test orientations were equally likely to be the same as and different from the corresponding memory items. On “new” trials, the orientation was always perpendicular to the original orientation of the memory item (90° apart). Note, this manipulation rendered mnemonic precision of retained VSTM representations largely irrelevant. That is, either coarse-grain or fine-grained VSTM representation for the test orientation is sufficient for detecting the change between memory and test (for extended discussion, see Xie and Zhang, 2017). Each participant completed 120 trials that were split into three experimental blocks. Responses from this task were fit with the DTSD model, yielding separate estimates of item and configural VSTM encoding for each participant. The details of the DTSD model (e.g., the equations and the theoretical interpretation of the model parameters) and the model fitting procedure were provided in Xie and Zhang (2017), and are thus omitted here.

In the Le Grand face task (Figure 1B), each trial began with an 800-ms fixation, followed by sequential presentations of two composite faces with a 300-ms interstimulus interval. Each face was presented for 200-ms. Participants reported whether the top halves of the two sequentially presented faces were the same or different (same and different trials were equally likely), while ignoring the bottom halves, which were always different. Note, to fit the entire experiment within a 1-h session, a partial design was used here, as compared to the complete design that also includes the condition in which the bottom halves were the same (e.g., Richler et al., 2011). On half of the trials, either the top or bottom haves of the two faces were shifted horizontally to the left by half a face width (the misaligned condition, the top row in Figure 1C), whereas on the remaining half of the trials, the top and bottom haves of each face were properly aligned (the aligned condition, the bottom row in Figure 1C). The faces in the aligned condition and misaligned condition were presented within a 4.8° × 7.2° and 7.2° × 7.2° rectangular area, respectively. The same and different trials were randomly mixed within experimental blocks, whereas misaligned and aligned conditions were blocked with the order counterbalanced across participants. Participants were instructed to make a *Same*-or-*Different* decision specifically to the two top halves, while ignoring the bottom halves, by pressing button “s” for *Same* or button “d” for *Different* on a computer keyboard as quickly and accurately as possible once the second face appeared. Both response time (RT) and accuracy were recorded. Twenty-five *same* trials and 25 *different* trials were presented for each of the aligned and misaligned conditions, yielding 100 trials in total.

CFE of reaction time (RT) was calculated by subtracting the median RT for misaligned trials from the median RT for aligned trials (Le Grand et al., 2004; Konar et al., 2010). Note, only RTs from trials with correct responses were used in this analysis. CFE was also calculated on mean *d*′, a signal detection theory measure (Macmillan and Creelman, 2005), in the same way as CFE on RT (Konar et al., 2010; Richler et al., 2011).

The modified GFMT task is a two-interval forced choice task using face stimuli adopted from Burton and colleagues (Burton et al., 2010). In this task, 150 pairs of gray-scale front-view Caucasian faces, subtending 5° × 7° of visual angel, were randomly selected from the GFMT set (see Burton et al., 2010 for details). Half of these pairs had matching identities and the other half had different identities. On each trial, two brief displays of faces (17-ms each) were presented sequentially with a 400-ms interstimuli interval. Note, this was different from the original GFMT in which the two faces were presented side by side simultaneously. The sequential presentation in the present study was to match the sequential presentations of face stimuli in the Le Grand task. Participants judged whether the two faces had the same identity or different identities while ignoring visual features that were irrelevant for identities. For example, the two faces with the matching identity in Figure 1D had subtle differences in contrasts, hairstyles, face contours, viewing angles, etc. This variance in identity-independent visual features is to ensure participants encode face identities across different views, which mimics faces recognition in natural vision (Burton et al., 2010).

Grubbs’ test (Grubbs, 1969) was conducted to detect potential outliers in all measures, although no outlier was identified in the present data, leading to zero outlier rejection.

## Results and Discussion

All three tasks were performed with reasonable accuracy. The change detection task yielded an average accuracy of 75% (72% 77%) [mean (95% confidence interval)] and average capacity (assessed as Cowan’s K, Cowan et al., 2005) of 2.48 (2.23 2.72). For the Le Grand face task, accuracy was averaged at 85% (83% 87%). More importantly, RTs on correct trials were significantly faster on misaligned trials than aligned trials [*t*(45) = 4.23, *p* < 0.001, Cohen’s *d* = 0.63, Bayes factor = 211.96], indicating more interference on aligned trials (and hence significant CFE on RT). Although CFE on *d*′ was significant [*t*(45) = 4.69, *p* < 0.001, Cohen’s *d* = 0.70, Bayes factor = 818.14], it was not significantly correlated with any measures in the present study (*p*’s > 0.30) and was thus not discussed further. For the modified GFMT discrimination task, accuracy was averaged at 78% (76% 80%).

**Figure 2:**
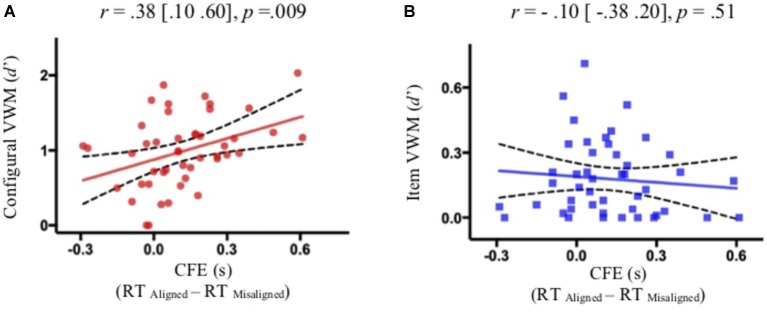
The significant correlation between holistic face encoding (CFE) and configural information retained in VSTM **(A)**, but not between holistic face encoding (CFE) and item information retained in VSTM **(B)**. The solid and broken lines represent linear regression fits and their 95% confidence intervals, respectively.

**Figure 3:**
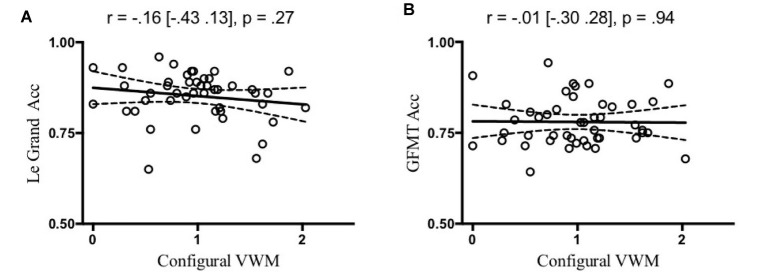
The correlation between configural VWM and Le Grand face task accuracy **(A)** and GFMT task accuracy **(B)** was not significant. The solid and broken lines represent linear regression fits and their 95% confidence intervals, respectively.

Of central importance, participants with more holistic face processing in the Le Grand face task had larger configural encoding in VSTM (Figure 2A), but comparable item encoding (Figure 2B), as compared to participants with less holistic face processing. That is, holistic face processing assessed as CFE significantly correlated with configural encoding [Pearson correlation: *r* = 0.38 (0.10 0.60), *p* = 0.009; Spearman’s rank-order correlation for non-Gaussian distribution in RT: *r* = 0.37 (0.08 0.60), *p* = 0.011], but not with item encoding [Pearson correlation: *r* = −0.10 (−0.38 0.20), *p* = 0.512; Spearman’s rank-order correlation: *r* = −0.10 (−0.39 0.20), *p* = 0.495]. Additionally, a multivariate regression analysis suggested that CFE significantly predicted configural VWM [*β* = 0.95 (0.25 1.64), *p* = 0.009] but not item VWM [*β* = −0.090 (−0.37 0.19), *p* = 0.512]. Critically, the correlation between CFE and configural encoding was significantly greater than the correlation between CFE and item encoding (*z* = 2.09, *p* = 0.018, one-tailed), based on a Fisher’s *r* to *z* transformation one-tailed test of correlated correlation (Meng et al., 1992). The relationship between configural VSTM and holistic face processing seems to be specific in that VSTM configural encoding did not significantly correlate with overall face processing assessed as the accuracy of the Le Grand face task (Figure 3A) [Pearson correlation: *r* = −0.16 (−0.43 0.13), *p* = 0.27; Spearman’s rank-order correlation: *r* = −0.25 (−0.51 0.06), *p* = 0.10] or the accuracy of the modified GFMT (Figure 3B) [Pearson correlation: *r* = −0.01 (−0.30 0.28), *p* = 0.94; Spearman’s rank-order correlation: *r* = −0.08 (−0.37 0.23), *p* = 0.61].

The lack of significant correlation between CFE and the overall face matching ability assessed as the accuracy of 2IFC discrimination task using GFMT face stimuli, although consistent with some previous findings (e.g., Konar et al., 2010), could simply result from the partial design (Richler et al., 2011). As a result, the present study was not optimal for assessing the relationship between configural face encoding and face recognition, which is beyond the scope of the present study.

Interestingly, CFE was significantly correlated with the overall performance of the VSTM change detection task [Accuracy, Pearson correlation: *r* = 0.30 (0.01 0.54), *p* = 0.04; Spearman’s rank-order correlation: *r* = 0.31 (0.01 0.56), *p* = 0.04; Capacity, Pearson correlation: *r* = 0.30 (0.01 0.55), *p* = 0.04; Spearman’s rank-order correlation: *r* = 0.31 (0.02 0.56), *p* = 0.03], potentially driven by the significant correlation between the CFE and VSTM configural encoding. The CFE could be considered as the magnitude of distractor processing in a way similar to the flanker compatibility effect (Zhang and Luck, 2014) given that both effects reflect how much distracting information (flanker distractor letters in the flanker task or bottom face halves in the Le Grand composite-face task) is processed. However, the positive correlation between VSTM capacity and the CFE seems to be inconsistent with the load theory of attention which predicts that higher WM capacity (equivalent to low cognitive load) would reduce distractor processing (for a recent review, see de Fockert, 2013; but see, Konstantinou and Lavie, 2013; Zhang and Luck, 2014). In other words, individuals with lower working memory capacities process distractors in an equivalent manner as higher working memory load conditions in tasks investigating the load theory of attention (de Fockert, 2013). In the present study, higher VSTM accuracy and capacity predicted a higher CFE, which is indicative of greater distractibility in the aligned face condition compared to the misaligned face condition. This inconsistency may result from the holistic nature of face processing in the Le Grand task. Further research is needed to directly compare effects of cognitive load on CFE and flanker compatibility effects.

## General Discussion

Given the hierarchical nature of visual representations in natural vision, it is essential for VSTM to retain hierarchical representations such as item and configural information. The present study assessed configural encoding and item encoding in VSTM for orientation using ROC modeling of change detection performance and assessed holistic face processing using the Le Grand CFE. We found that configural encoding, but not item encoding, for orientations in VSTM significantly correlated with holistic processing in face discrimination in that participants with more configural VSTM also showed larger holistic face encoding. These findings add to the growing literature on the effects of perceptual organization on VSTM.

In addition to its selective correlation with holistic face processing, configural information retained in VSTM could also be experimentally dissociated from item information retained in VSTM using selective experimental manipulations (Xie and Zhang, 2017). Together these findings are essential for scaling up models for VSTM to account for both item information and global stimulus structures (Kroll et al., 1996; Reinitz et al., 1996), especially for natural stimuli (Greene and Oliva, 2009; Brady et al., 2011; Brady and Tenenbaum, 2013).

The Dual Trace Signal Detection model for VSTM is mathematically equivalent to the Dual-Process Signal Detection (DPSD) of Recognition memory (Yonelinas et al., 1999). According to DPSD model, recognition is based on a high-threshold discrete recollection and a continuous familiarity (modeled as *d*′). The *d*′ component shared across the two models seems to suggest some relationship between configural encoding and familiarity. For instance, configural information may be more important for supporting familiarity than item information. Consequently, face inversion that profoundly impairs holistic face processing (for review, see Maurer et al., 2002) can significantly reduce familiarity (Yonelinas et al., 1999). In addition, processing of higher level representations (e.g., summary statistics and configural information) is more automatic than processing of discrete item representations (e.g., Alvarez and Oliva, 2009), consistent with the proposal that familiarity is less controlled than recollection (Yonelinas and Jacoby, 1996; Yonelinas, 2002).

In the present study, configural VSTM correlates with holistic face encoding (CFE), but not with overall face discrimination (the 2IFC using GFMT stimuli). The latter finding was not necessarily inconsistent with a previously reported correlation between VSTM and face identification (Megreya and Burton, 2006). The face identification task in Megreya and Burton’s (2006) study used a procedure in which the participants match one target face to one of 10 possible faces. This procedure could exert a high demand on VSTM. When demand on VSTM was significantly reduced using a two-alternative force choice task with two simultaneously presented faces in the GFMT, no significant correlation was found between face matching and VSTM (Burton et al., 2010). It is thus possible that a robust relationship between VSTM and face processing could manifest to face recognition tasks with high working memory load.

The present study has focused on how configural processing is shared between face perception and VSTM for orientation. This issue is orthogonal to the role of holistic processing in face processing (Konar et al., 2010; Richler et al., 2011; Gold et al., 2012; DeGutis et al., 2013), which is beyond the scope of the present study. While Konar et al. (2010) used a full factorial design in which the bottom face halves were equally likely to be the same or different the current study used a partial design. The partial design may be suboptimal in that it could induce systematic biases of reporting “same,” which may account for some conflicting findings on the effects of holistic encoding in face identification (Konar et al., 2010; Richler et al., 2011). However, it is less of an issue in the present study given a significant relationship was found between holistic face processing and configural VSTM. Nonetheless, it is important for future research to replicate this finding using a full factorial design in the Le Grand CFE task. It will also be interesting to link item and configural encoding in VSTM to part-based encoding and holistic encoding in processing of facial expressions (Tanaka et al., 2012). Furthermore, a stronger test of the relationship between holistic face processing and configural encoding in VSTM is to assess whether holistic face processing can predict increased VSTM performance in VSTM from a condition where configural encoding is minimized (Xie and Zhang, 2017) to conditions where configural encoding is prominent (Xie and Zhang, 2017).

In summary, the present study, using an individual differences approach, has demonstrated that VSTM for configural information can be accounted for by holistic face perception, providing preliminary evidence that configural encoding in VSTM may result from configural encoding in perception.

## Data Availability Statement

The datasets generated for this study are available on request to the corresponding author.

## Ethics Statement

The studies involving human participants were reviewed and approved by the University of California, Riverside IRB. The patients/participants provided their written informed consent to participate in this study.

## Author Contributions

WZ designed the study, collected data, and conducted data analysis. Both authors contributed to manuscript preparation.

### Conflict of Interest

The authors declare that the research was conducted in the absence of any commercial or financial relationships that could be construed as a potential conflict of interest.
